# Niemann-Pick C1 (NPC1)/NPC1-like1 Chimeras Define Sequences Critical for NPC1’s Function as a Filovirus Entry Receptor

**DOI:** 10.3390/v4112471

**Published:** 2012-10-26

**Authors:** Anuja Krishnan, Emily Happy Miller, Andrew S. Herbert, Melinda Ng, Esther Ndungo, Sean P. Whelan, John M. Dye, Kartik Chandran

**Affiliations:** 1 Department of Microbiology and Immunology, Albert Einstein College of Medicine, 1300 Morris Park Ave, Bronx, NY 10461, USA; Email: anuja@immindia.org (A.K.), emily.miller@med.einstein.yu.edu (E.M.), melinda.ng@phd.einstein.yu.edu (M.N.), esther.ndungo@phd.einstein.yu.edu (E.N.); 2 US Army Medical Research Institute of Infectious Diseases, 1425 Porter St, Fort Detrick, MD 21702, USA; Email: andrew.s.herbert@us.army.mil (A.H.), john.m.dye1@us.army.mil (J.D.); 3 Department of Microbiology and Immunobiology, 200 Longwood Ave, Harvard Medical School, Boston, MA 02115, USA; Email: sean.whelan@hms.harvard.edu; 4 Institute of Molecular Medicine, Okhla Industrial Estate, Phase III, New Delhi 110020, India; Email: anuja@immindia.org

**Keywords:** Ebola virus, Marburg virus, filovirus, viral entry, Niemann-Pick C1, NPC1, Niemann-Pick C1-like1, NPC1L1, host factor, viral receptor

## Abstract

We recently demonstrated that Niemann-Pick C1 (NPC1), a ubiquitous 13-pass cellular membrane protein involved in lysosomal cholesterol transport, is a critical entry receptor for filoviruses. Here we show that Niemann-Pick C1-like1 (NPC1L1), an NPC1 paralog and hepatitis C virus entry factor, lacks filovirus receptor activity. We exploited the structural similarity between NPC1 and NPC1L1 to construct and analyze a panel of chimeras in which NPC1L1 sequences were replaced with cognate sequences from NPC1. Only one chimera, NPC1L1 containing the second luminal domain (C) of NPC1 in place of its own, bound to the viral glycoprotein, GP. This engineered protein mediated authentic filovirus infection nearly as well as wild-type NPC1, and more efficiently than did a minimal NPC1 domain C-based receptor recently described by us. A reciprocal chimera, NPC1 containing NPC1L1’s domain C, was completely inactive. Remarkably, an intra-domain NPC1L1-NPC1 chimera bearing only a ~130-amino acid N–terminal region of NPC1 domain C could confer substantial viral receptor activity on NPC1L1. Taken together, these findings account for the failure of NPC1L1 to serve as a filovirus receptor, highlight the central role of the luminal domain C of NPC1 in filovirus entry, and reveal the direct involvement of N–terminal domain C sequences in NPC1’s function as a filovirus receptor.

## 1. Introduction

Ebola virus (EBOV) and Marburg virus (MARV) are associated with fulminant and highly lethal outbreaks of hemorrhagic fever for which no approved vaccines or treatments exist. These viruses are members of the family *Filoviridae* of enveloped viruses with nonsegmented negative-strand RNA genomes (filoviruses) [1]. Filoviruses encode a single entry glycoprotein, GP, which forms trimeric spikes at the viral surface [2,3]. The GP precursor is post-translationally cleaved by the pro-protein convertase furin within the Golgi compartment of virus-producer cells, yielding two subunits, GP1 and GP2. GP1 binds to cellular receptors and controls GP2 conformation; GP2 catalyzes fusion between viral and cellular membranes.

Viral particles attach to host cells through interactions with a variety of cell-surface molecules [4,5,6], and are then internalized and delivered to late endosomes [7,8,9]. Here, endosomal cysteine proteases cleave GP1 to remove heavily glycosylated C–terminal sequences, generating an entry intermediate comprising an N–terminal GP1 fragment and GP2 [10,11,12,13]. We recently showed that cleaved GP must bind to Niemann-Pick C1 (NPC1), a 13-pass transmembrane protein resident in late endosomes and implicated in lysosomal cholesterol transport [14]. Events in entry downstream of GP-NPC1 binding remain obscure, but they must culminate in the GP2-mediated fusion of viral and cellular membranes and cytoplasmic delivery of the viral nucleocapsid. The GP conformational changes that drive membrane merger are triggered by an undefined host stimulus [15,16,17].

The authors of this manuscript [18] and other researchers [19] recently established that NPC1 is an essential host factor for filovirus entry, infection, and pathogenesis, and a critical viral receptor [14]. NPC1 is a ubiquitous housekeeping protein that plays a crucial role in the regulated efflux of cholesterol from lysosomes [20,21,22], and its loss in humans causes Niemann-Pick type C disease, a fatal lysosomal storage disorder [23]. This cellular function of NPC1 is dispensable for filovirus entry [14,18,19], which instead requires the direct association of cleaved GP with the second major luminal domain of NPC1, domain C [14]. Moreover, synthetic membrane proteins containing NPC1 domain C possess viral receptor activity, indicating that it is sufficient for filovirus entry [14]. These findings notwithstanding, the greatly reduced levels of viral infection obtained with minimal domain C-containing receptors and other NPC1 deletion mutants, relative to the WT protein, suggest supporting roles for additional NPC1 sequences in filovirus entry [14].

Davies and co-workers [24] identified a protein in vertebrates with substantial homology (~40% amino acid sequence identity) to NPC1. This protein, NPC1-like1 (NPC1L1), closely resembles NPC1 in overall architecture, possessing 13 transmembrane proteins and three large luminal domains (A, C, and I) [24,25]. Moreover, NPC1L1, like NPC1, participates in cellular cholesterol metabolism (reviewed in [26]). Unlike NPC1, however, NPC1L1 is expressed only in gut epithelial cells (in all mammals examined) and liver hepatocytes (in humans and non-human primates). The two proteins also exhibit functional differences: NPC1 is involved in cholesterol efflux from lysosomes, but NPC1L1 mediates cholesterol absorption from the extracellular compartment. Here, we show that NPC1L1, recently implicated in cell entry by hepatitis C virus [27], completely lacks filovirus receptor activity. We exploit this observation, together with the structural similarity between these two proteins, to generate a panel of chimeras between NPC1 and NPC1L1. Analysis of this panel for viral receptor activity yielded both gain-of-function and loss-of-function phenotypes, allowing the identification of sequences in NPC1 that play roles in filovirus entry, and providing an explanation for the failure of NPC1L1 to serve as a filovirus receptor.

## 2. Results and Discussion

### 2.1. Human NPC1-like1 (NPC1L1) cannot mediate filovirus entry

**Figure 1:**
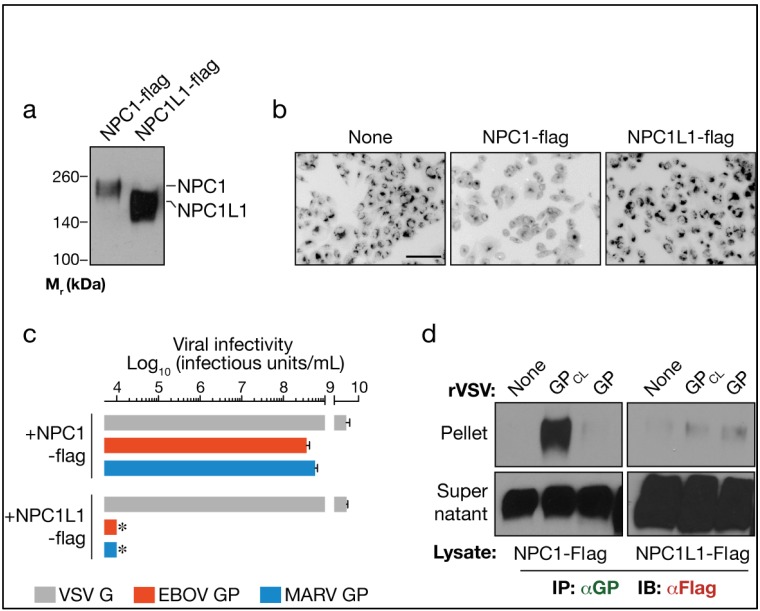
Human Niemann-Pick C1-like1 (NPC1L1) lacks filovirus receptor activity. (a) NPC1-null Chinese hamster ovary (CHO) CT43 cells were engineered to express human NPC1-flag or NPC1L1-flag. Protein expression was detected by immunoblotting with an anti-flag antibody. M_r_, relative molecular weight in kilodaltons (kDa). (b) Efflux of lysosomal cholesterol mediated by NPC1-flag and NPC1L1-flag in CT43 cells was determined by filipin staining and fluorescence microscopy. Scale bar, 20 μm. (c) Infection of CT43 cells expressing NPC1-flag or NPC1L1-flag by vesicular stomatitis virus (VSV) pseudotypes bearing VSV G or filovirus glycoproteins. Asterisks indicate data points at or below the limit of detection. (d) Co-immunoprecipitation (co-IP) of NPC1 and NPC1L1 by Ebola virus (EBOV) GP. Magnetic beads coated with GP-specific monoclonal antibody KZ52 were incubated with detergent extracts containing no virus (None), uncleaved VSV-GP, or cleaved VSV-GP_CL_. Control or glycoprotein-decorated beads were mixed with cell extracts containing NPC1-flag or NPC1L1-flag. Beads were then retrieved and flag-tagged proteins in the immune pellets and supernatants were detected by immunoblotting (IB).

**Figure 2:**
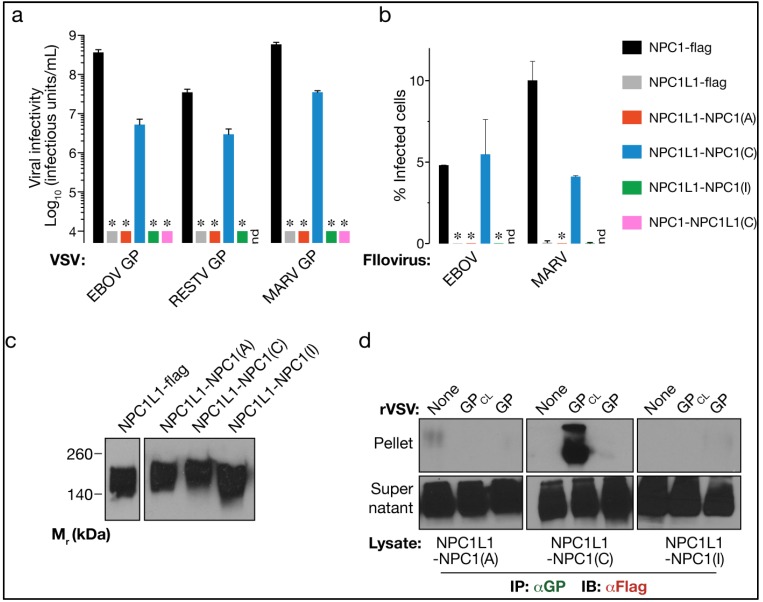
NPC1’s domain C converts NPC1L1 into a highly effective filovirus receptor. (a-b) Infection of CT43 cells expressing NPC1-flag, NPC1L1-flag, or the indicated flag-tagged NPC1L1-NPC1 chimeras by VSV pseudotypes bearing filovirus glycoproteins (a) or authentic filoviruses. nd, not determined. (b). Asterisks indicate data points at or below the limit of detection. (c) Expression of NPC1L1-flag and the indicated flag-tagged NPC1L1-NPC1 chimeras in CT43 cells was detected by immunoblotting with an anti-flag antibody. (d) Co-immunoprecipitation of NPC1L1-NPC1 chimeras by EBOV GP was performed as described in Fig. 1d.

To determine if NPC1L1 can mediate filovirus entry, we used an NPC1-mutant cell line described by Chang and co-workers (Chinese hamster ovary [CHO] CT43) [21]. The hamster NPC1 gene in CT43 cells contains a 116-bp deletion in exon 19 that creates a frameshift and leads to premature translational termination after 933 amino acids; however, no truncated NPC1 polypeptide was detected in these cells [28](T.Y. Chang, personal communication). Concordantly, our previous [14,18] and current (Figs. 1-2) findings indicate that CT43 cells are completely resistant to filovirus entry and infection, but become highly susceptible when wild-type NPC1 is ectopically expressed. Taken together, these observations confirm that the CT43 line is functionally NPC1-null. 

We stably expressed a Flag epitope-tagged form of NPC1L1 in CT43 cells (Fig. 1a), and assessed their capacity to mediate lysosomal cholesterol transport and viral infection (Fig. 1). As we showed previously, the parental CT43 cells accumulated lysosomal cholesterol [21] (Fig. 1b) and were completely refractory to infection by vesicular stomatitis virus (VSV) pseudotypes bearing filovirus glycoproteins (VSV-GP-EBOV/MARV) (not shown). Also as expected, Flag-tagged WT NPC1 (NPC1-flag) reversed both cholesterol transport and viral infection defects (Fig. 1b-c). In contrast to their counterparts containing NPC1-flag, CT43-NPC1L1-flag cells accumulated lysosomal cholesterol and remained resistant to rVSV-GP infection (Fig. 1b-c). Therefore, despite its extensive sequence similarity to NPC1 and high level of expression in CT43 cells (Fig. 1a), NPC1L1-flag is inactive at both cholesterol clearance and filovirus entry (Fig. 1b-c).

### 2.2. NPC1L1 cannot bind to cleaved EBOV GP

We recently demonstrated that in vitro-cleaved, but not uncleaved EBOV GP could bind directly to human NPC1, and that GP-NPC1 interaction is required for filovirus entry [14]. To determine the capacity of NPC1L1 to interact with GP, we tested if intact and/or cleaved EBOV GP (GP_CL_) could retrieve NPC1L1-flag from detergent extracts of CT43 cells (Fig. 1d). Briefly, rVSV-GP-EBOV particles were solubilized in a nonionic detergent-containing buffer, and the GP protein in these extracts was captured by magnetic beads coated with the GP-specific monoclonal antibody KZ52 [28]. The GP-decorated beads were then incubated with detergent extracts of CT43 cells containing either NPC1-flag or NPC1L1-flag. As observed previously [14], GP_CL_, but not GP, could retrieve NPC1-flag from cell extracts. However, neither protein could capture NPC1L1-flag (Fig. 1d). Therefore, NPC1L1 possesses little or no capacity to bind to EBOV GP. Taken together with its failure to support viral entry (Fig. 1b-c), these findings provide evidence that NPC1L1 is not an entry receptor for filoviruses.

### 2.3. NPC1’s domain C renders NPC1L1 highly competent to bind to EBOV GP and mediate filovirus entry

We showed previously that domain C, the second major luminal domain of NPC1, is critical for filovirus entry and binds directly to an in vitro-cleaved form of EBOV GP, GP_CL_ [14]. However, because synthetic membrane proteins engineered to contain NPC1’s domain C reconstituted viral infection only inefficiently in NPC1-null cells (to ~0.01% of WT NPC1), our prior studies did not rule out the potential involvement of other NPC1 sequences to filovirus entry. Here, we took an alternative approach to assess the contribution of each major luminal domain of NPC1 in the context of the full-length protein: we replaced sequences in NPC1L1 with their counterparts from NPC1, and examined the capacity of the resulting chimeras to support viral infection and bind to cleaved EBOV GP. NPC1-null CT43 cells were engineered to stably express chimeras containing domains A, C, or I from NPC1 in an NPC1L1 background (see Materials and Methods for details). All three chimeras (and WT NPC1L1) were found to be expressed at similar levels (Fig. 2c). These cells were then challenged with VSV-GP particles bearing glycoproteins from EBOV, Reston virus (RESTV), and MARV (Fig. 2a). NPC1L1-NPC1(C), an NPC1L1 chimera bearing NPC1’s domain C, was highly competent to mediate filovirus entry. By contrast, NPC1L1 chimeras bearing NPC1’s domains A or I were completely inactive, as was an inverse chimera bearing NPC1L1’s domain C in an NPC1 background (NPC1-NPC1L1(C); Fig. 2a). Importantly, similar results were obtained in infections with authentic EBOV and MARV under BSL-4 containment (Fig. 2b).

We next tested the capacities of the NPCL1-NPC1 chimeras to bind to EBOV GP and GP_CL_ in vitro, as described in Fig. 1d. Only the chimera bearing NPC1’s domain C was captured from CT43 detergent extracts by GP_CL_ (Fig. 2d). Taken together, these findings corroborate our previous work identifying domain C as the sole indispensable requirement in NPC1 for GP-NPC1 binding and filovirus entry. They also indicate that NPC1L1 cannot act as a filovirus receptor at least in part because its divergent domain C cannot recognize and bind to EBOV GP.

### 2.4. NPC1L1 sequences greatly enhance the capacity of NPC1’s domain C to serve as a filovirus entry receptor

**Figure 3:**
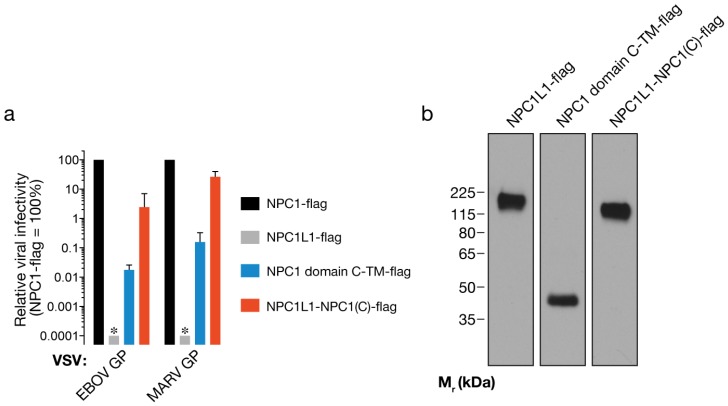
NPC1L1 sequences greatly enhance the capacity of NPC1’s domain C to mediate filovirus entry. (a) Infection of CT43 cells expressing NPC1-flag, NPC1L1-flag, NPC1 domain C fused to NPC1’s transmembrane domain (NPC1 domain C-TM-flag), or the chimera NPC1L1-NPC1(C)-flag by VSV pseudotypes bearing filovirus glycoproteins. Asterisks indicate data points at or below the limit of detection. (b) Expression of the indicated flag-tagged proteins in CT43 cells was detected by immunoblotting with an anti-flag antibody. Samples in Figs. 3b and 4c were resolved on the same SDS-polyacrylamide gel.

Minimal synthetic membrane proteins containing NPC1’s domain C (NPC1 domain C-TM) are competent to mediate filovirus entry, but at greatly reduced levels relative to WT NPC1 [14]. To evaluate the effect of flanking sequences from NPC1L1 on the viral receptor activity of NPC1 domain C, we compared the infectivities of VSV particles bearing EBOV or MARV GP in cells expressing NPC1 domain C-TM or NPC1L1-NPC1(C) (Fig. 3a-b). We found that insertion of NPC1’s domain C into the divergent NPC1L1 sequence conferred a dramatic (~100-fold) increase in its capacity to mediate filovirus entry, with infection by VSV-MARV GP in NPC1L1-NPC1(C)-expressing cells approaching levels measured in cells expressing WT NPC1 (Fig. 3a). These findings suggest two non-mutually exclusive possibilities: first, NPC1L1 sequences provide the structural scaffold necessary for optimal folding and/or display of NPC1’s domain C on the endosomal membrane; second, NPC1L1 sequences outside of domain C can substitute for cognate sequences in NPC1 in directly mediating filovirus entry, despite the marked evolutionary divergence of NPC1L1 and NPC1.

### 2.5. An N–terminal region of NPC1’s domain C can confer filovirus receptor activity upon NPC1L1

**Figure 4:**
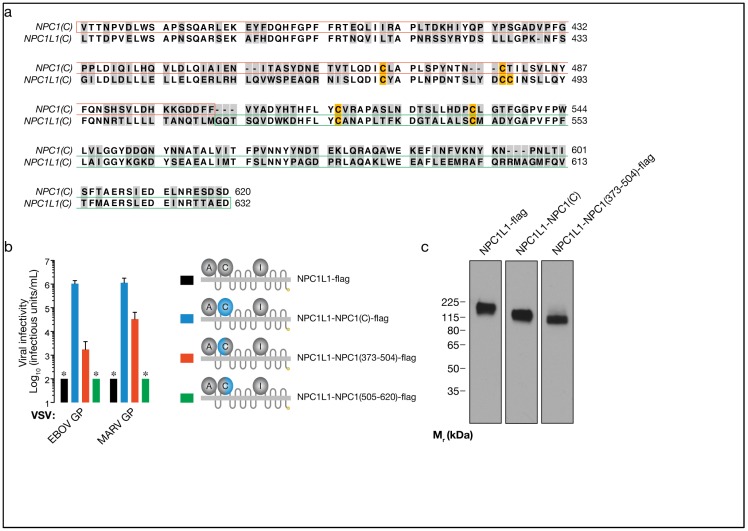
N-terminal sequences from NPC1 domain C render NPC1L1 competent to mediate filovirus entry. (a) Alignment of domain C amino acid sequences from human NPC1 and NPC1L1. Sequence differences are shaded in gray. Sequences exchanged between domains are outlined in red and green. Cysteines are highlighted in yellow. (b) Infection of CT43 cells expressing NPC1L1-flag, NPC1L1-NPC1(C)-flag, or NPC1L1-NPC1 intra-domain C chimeras by VSV pseudotypes bearing filovirus glycoproteins. Asterisks indicate data points at or below the limit of detection. (c) Expression of the indicated flag-tagged proteins in CT43 cells was detected by immunoblotting with an anti-flag antibody. Samples in Figs. 3b and 4c were resolved on the same SDS-polyacrylamide gel.

Finally, we reasoned that the sequence (and putative structural) similarities between the C domains of NPC1 and NPC1L1 (Fig. 4a), may afford the construction of additional gain-of-function chimeras, but this time within domain C itself. Accordingly, we generated two NPC1L1-NPC1 chimeras in which an N-terminal or a C-terminal portion of NPC1L1 was replaced with the corresponding sequence from NPC1 (NPC1L1-NPC1(373-504)-flag and NPC1L1-NPC1(505-620)-flag, respectively) (Fig. 4b-c). We found that, despite their similar levels of expression in CT43 cells, the two chimeras differed dramatically in their capacity to support filovirus entry. NPC1L1 bearing N-terminal domain C sequences from NPC1 could mediate viral entry at substantial levels, comparable to that afforded by the minimal NPC1 domain C-TM receptor; in contrast, the chimera bearing C-terminal domain C sequences from NPC1 was completely inactive. These findings reveal a critical role for N-terminal sequences within domain C in NPC1’s filovirus receptor function. Moreover, given the failure of NPC1L1’s domain C to bind to EBOV GP and support infection (Fig. 1), our observations with this gain-of-function mutant indicate that N-terminal domain C sequences in NPC1 are directly involved in filovirus entry, possibly at the level of GP binding.

## 3. Experimental Section

### 3.1. Cell culture

NPC1-null (CT43) Chinese hamster ovary (CHO) fibroblasts were maintained in DMEM-Ham’s F-12 medium (50-50 mix) supplemented with 10% FCS, L-glutamine, and penicillin–streptomycin. Vero African grivet monkey kidney cells and 293T human embryonic kidney cells were maintained in DMEM supplemented with 10% FCS, L-glutamine, and penicillin–streptomycin. All mammalian cell lines were maintained in a humidified 5% CO2 incubator.

### 3.2. NPC1 and NPC1L1 constructs

Flag-tagged NPC1L1-NPC1 chimeras were constructed by splice overlap extension PCR [29] and subcloned into the *BamHI* and *SalI* restriction sites of the pBABE-puro retroviral vector [30]. NPC1L1-NPC1(A) contains amino acid residues NPC1 residues 1-266 and NPC1L1 residues 288-1332; NPC1L1-NPC1(C) contains NPC1L1 residues 1-369, NPC1 residues 367-620, and NPC1L1 residues 633-1332; NPC1L1-NPC1(I) contains NPC1L1 residues 1-868, NPC1 residues 859-1097, and NPC1L1 residues 1113-1332. All constructs were verified by automated DNA sequencing.

CT43 cell populations stably expressing human NPC1-flag, NPC1L1-flag, and NPC1L1-NPC1 chimeras were generated by retroviral transduction as described previously [14].

### 3.3. Viruses and infections

VSV pseudotypes bearing glycoproteins derived from VSV, EBOV, RESTV, and MARV were generated as described previously [31]. The authentic filoviruses EBOV-Zaire 1995 and MARV-Ci67 used in this study have been described previously [32,33]. VSV particles containing GP_CL_ were generated by incubating rVSV-GP-EBOV with thermolysin (200 μg/mL) for 1 h at 37˚C. The protease was inactivated by addition of phosphoramidon (1 mM), and reaction mixtures were used immediately.

Infectivities of VSV pseudotypes were measured by manual counting of eGFP-positive cells using fluorescence microscopy at 16-24 h post-infection.

Cells were exposed to authentic viruses at an MOI of 3 for 1 h. Viral inoculum was then removed and fresh culture media was added. At 48 h post-infection, cells were fixed with formalin, and blocked with 1% bovine serum albumin. Detection and quantitation of infected cells and uninfected controls was done as described previously [14].

### 3.4. NPC1/NPC1L1-containing cell extracts for GP-binding assays

Extracts were prepared as described previously [14]. CT43 cells expressing NPC1-flag, NPC1L1-flag, and NPC1L1-NPC1 chimeras were washed with PBS, and packed cell pellets were lysed by incubation at 4˚C with NTE-CHAPS buffer (10 mM Tris[pH 7.5], 140 mM NaCl, 1 mM EDTA, 0.5% vol/vol 3-[(3-cholamidopropyl)dimethylammonio]-1-propanesulfonate)) supplemented with a protease inhibitor cocktail (Roche). Typically, 1 mL buffer was used to lyse 2×10^7^ cell-equivalents. To promote cell lysis, cell suspensions were probe-sonicated (lowest setting, 5 pulses of 5 sec each) in an ice-water bath. Lysates were cleared by centrifugation at 14,000 ×g for 10 min, and supernatants were used immediately.

### 3.5. GP-NPC1 co-immunoprecipitation (co-IP) assays

Co-IP assays were carried out as described previously [14]. Protein G-coated magnetic beads (20 μL/reaction; Spherotech) were incubated with the GP-specific monoclonal antibody KZ52 (5 μg) [28] for 1 h, washed to remove unbound antibody, and then added to uncleaved or in vitro-cleaved VSV-GP-EBOV particles (5 μL concentrated virus; 10^7^–10^8^ infectious units) in NTE-CHAPS buffer. Bead-virus mixtures were incubated for 2 h at room temperature, and then added to crude detergent extracts of CHO CT43 cells expressing flag-tagged NPC1 or related proteins (2×10^6^ cell-equivalents). After overnight incubation with mixing at 4˚C, beads were retrieved with a magnet, extensively washed with NTE-CHAPS, and heated in Laemmli sample buffer to elute bound proteins. Solubilized proteins were subjected to SDS-polyacrylamide gel electrophoresis, and flag-tagged proteins were detected by immunoblotting with an anti-flag antibody (Sigma Aldrich). Typically 50-100% of each pellet sample and 5-10% of each supernatant were loaded on gels.

## 4. Conclusions

Our findings afford the following conclusions: (1) They firmly establish that NPC1, but not its structurally- and functionally-related paralog NPC1L1, can serve as a filovirus receptor by binding directly to the viral glycoprotein. (2) They confirm the indispensable role of domain C, NPC1’s second major luminal domain, for filovirus receptor activity. (3) They reveal that NPC1L1 is inactive as a filovirus receptor because its divergent domain C cannot bind to the viral glycoprotein. (4) Non-domain C sequences derived from NPC1L1 substitute essentially fully for their counterparts from NPC1, arguing that structural and/or functional elements conserved among NPC1 gene family members play key roles in mediating filovirus entry. Additional studies to identify these conserved sequences in NPC1 and NPC1L1 and define their roles in filovirus entry are therefore warranted. (5) An 131-amino acid sequence approximating the N-terminal half of NPC1 domain C confers filovirus receptor activity on NPC1L1, suggesting that amino acid residues within this sequence directly contact GP during filovirus entry. Our current work is aimed at further delineating sequences in domain C that play direct roles in NPC1’s filovirus receptor function.
